# A Standardized Transcutaneous Electric Acupoint Stimulation for Relieving Tobacco Urges in Dependent Smokers

**DOI:** 10.1093/ecam/nen074

**Published:** 2011-06-05

**Authors:** Caroline Lambert, Ivan Berlin, Tat-Leang Lee, Siew Wan Hee, Audrey S. L. Tan, David Picard, Ji Sheng Han

**Affiliations:** ^1^Moleac Pte Ltd, Helios Building #09-08, 11, Biopolis way, Singapore 138667, Singapore; ^2^Service de Pharmacologie, Groupe Hospitalier Universitaire Pitié-Salpêtrière-Faculté de médecine Paris 6-Inserm U677, Paris, France; ^3^Department of Anaesthesia, National University Hospital, 5 Lower Kent Ridge Road, Singapore 119074, Singapore; ^4^Clinical Trials and Epidemiological Sciences, National Cancer Centre, 11 Hospital Drive, Singapore 169610, Singapore; ^5^National Healthcare Group, 6 Commonwealth Lane, Level 6 GMTI Building, Singapore 149547, Singapore; ^6^Neuroscience Research Institute, Peking University, 38 Xue Yuan Road, Beijing 100083, China

## Abstract

The efficacy of acupuncture in smoking cessation, and its effect on the urge to smoke are unclear. We evaluated the effect of a standardized protocol of transcutaneous electric acupoint stimulations (TEAS) on alleviating the urge to smoke. Ninety-eight smokers were recruited in two double-blind studies. Participants abstained from smoking for 26 h, and were randomized to receive TEAS alternating between 2 and 100 Hz at four acupoints (LI4 and PC8, PC6 and TE5) at four different intensities (10, 5, Intermittent 5 or 0 mA). The urge to smoke was assessed by the Questionnaire of Smoking Urges (QSU-Brief). In Experiment 1, the 10 mA group (*n* = 20) was compared with the 5 mA group (*n* = 20); the increase in smoking urges did not differ significantly. Considering the possibility that 5 mA may be an active intervention, in Experiment 2, a true placebo (0 mA), and a proxy of placebo [Intermittent 5 mA (i5 mA)] were compared with 10 mA TEAS. In this experiment, 10 mA (*n* = 20) TEAS showed a tendency to alleviate smoking urges compared with 0 mA (*n* = 16), and i5 mA (*n* = 19) TEAS. Only when the data of smokers with Fagerstöm Test for Nicotine Dependence score ≥5 were analyzed that the difference between the 10 mA group and the control group (0 and i5 mA) became significant. Based on these preliminary findings, we conclude that TEAS applied on the skin may antagonize the increase in urge to smoke in abstinent-dependent smokers. It seems warranted to assess the efficacy of TEAS in smoking cessation clinical trials involving a larger population of dependent smokers.

## 1. Introduction

Several types of interventions, such as counseling [1], nicotine replacement therapies (NRT) [2], or psychotropic drugs such as bupropion [3] have shown efficacy in smoking cessation. Less-studied complementary interventions, including relaxation, acupuncture, electro-acupuncture, hypnosis, meditation, green tea, Vitamin C or E, are increasingly sought-after alternative therapies by smokers who wish to quit smoking [4]. However, their efficacy is yet to be confirmed. Uncontrolled studies have suggested that acupuncture might reduce the symptoms of nicotine withdrawal, and some high rates of initial success have been reported [5]. Many literature reviews of controlled trials of acupuncture for smoking cessation have been published [6–13], but their conclusions have been found to be inconsistent. A recent Cochrane review [14] concluded that despite the relatively high number [15] of studies, there was no consistent evidence that acupuncture was effective for smoking cessation. Firm conclusions could not be drawn because of methodological inadequacies in the studies, such as the failure to validate smoking cessation, inadequate details of randomization procedures and failure to minimize the investigators' influence on the subjects. It was thus concluded that further well-designed studies were justified.

Acupuncture can be applied in different ways. Manual needling is the classic method, and electro-acupuncture (EA), that is, electrical stimulation is introduced to the body via metallic needles inserted into the acupoints is the most commonly used modality today [16], which has a characteristic feature of highly reproducible stimulation parameters, and great savings in manpower. However, in the case of the treatment of drug dependence, there is often an urgent need for treatment when a craving episode suddenly occurs. It is almost impossible for the subject to find an acupuncturist within a few minutes before he/she is compulsively rushing for the drug. An alternative is to use skin electrodes to apply electrical stimulation on the acupoints by the patient himself/herself, with a method called transcutaneous electrical acupoint stimulation (TEAS).

A thorough study has been made to compare the effectiveness of EA and TEAS in producing antinociceptive effect in rats [17]. The results showed that TEAS was as effective as EA, if not more, in terms of its antinociceptive efficacy, and that both effects could be blocked by the opioid receptor antagonist naloxone to the same extent. In a recent study performed in humans on former heroin addicts, the video cue-induced craving assessed by the visual analog scale (VAS), as well as the accompanying changes of the autonomic function (increase of blood pressure and heart rate) was effectively controlled by the TEAS using the HANS unit [18].

While the effect of acupuncture has been the subject of increasing amount of research, the central activation pattern induced by acupuncture (increased production and release of morphine-like substances such as enkephalins, endorphins, dynorphins and other neurotransmitters) has only recently been clarified in animal model [16, 19], and in humans [20]. The parameters (intensity, frequency and locations of the acupoints) of EA for opioid addiction have been evaluated in considerable detail [18, 19, 21–24]. According to these findings, a specific standardized protocol of TEAS was established, and animal studies demonstrated that the effect of TEAS was very similar, if not more potent, than EA [17].

Acute cravings for cigarettes (i.e., the urge to smoke) have repeatedly been associated with relapses, and are identified as having a critical role in promoting smoking relapse [15]. Therefore, relieving the urge to smoke may be a major goal for demonstrating the potential clinical effect of a treatment on abstinence. It can thus be hypothesized that if TEAS decreases the urge to smoke, it can later be considered as a potential therapeutic tool, and can be assessed in a randomized controlled therapeutic trial of smoking cessation.

The aim of the present experiments was to evaluate the efficacy of a standardized protocol of TEAS in alleviating the urge to smoke in nicotine-dependent individuals, during a 26-h abstinence period. Two exploratory studies were performed at Singapore's National University Hospital (NUH). The first (Experiment 1) was a pilot study to investigate the efficacy of TEAS as a method for relieving acute cravings in a controlled situation, where smokers were not allowed to smoke [25]. This experiment was initiated in March 2005 to assess the feasibility, the acceptability by the subjects and the dropout rate. Based on the findings obtained in the first study, a second study (Experiment 2) was implemented in November 2005 to evaluate the effect of three different TEAS stimulations on the urge to smoke; this study included a true placebo (zero stimulation) and, a proxy of placebo (very weak stimulation). The TEAS device and the conditions of the outline were specially designed to ascertain the validity of double-blind; and a guess test was performed with both research subjects and investigators to validate the blinding, and to ascertain that the subjects and the investigators were unable to identify the placebo intervention.

## 2. Methods

### 2.1. Study Design

Two studies were performed consecutively. Both were single-center, randomized and double-blind pilot studies evaluating smoking urges in smokers with a Fagerström score higher than 4 (Fagerström Test for Nicotine Dependence, FTND [26]), while receiving several modalities of TEAS treatment during a 26-h abstinence period.

In the first study (Experiment 1), a total of 40 participants (38 males and 2 females) were recruited through the NUH clinical trial unit database (*n* = 23), and through street interviews (*n* = 17) and they were randomized by using the web randomization software (http://www.randomization.com/) into two parallel groups: 20 subjects receiving 10 mA TEAS stimulations and 20 subjects receiving 5 mA TEAS stimulations. The intensity of the latter corresponds to a threshold value of tingling sensation to ensure double-blinding as much as possible. At this level of stimulation, the subject feels that he/she is receiving treatment, but very little analgesic effect could be observed according to our earlier human studies on pain modulation. However, the concern that the continuous 5 mA TEAS also represents a form of active treatment cannot be rejected.

For the second study (Experiment 2), a total of 58 participants (49 males and 9 females) were recruited through street interviews and randomized (via http://www.randomization.com/) into three groups: participants were given either continuous TEAS treatment at 10 mA (*n* = 21), an intermittent (3 min on and 7 min off) TEAS at 5 mA (i5 mA) (*n* = 20) or a placebo TEAS with no electrical stimulation (0 mA) (*n* = 17). The i5 mA intensity represents an extremely weak, but still sensible stimulation.

Subjects in both groups received four TEAS sessions: one session was performed on Day 1 (18:00 h in the non-abstinent state and three sessions on Day 2 (08:30, 12:30 and 16:30 h) while abstaining from smoking.

### 2.2. Participants

Participants were prescreened, to ensure that they had an FTND score equal to or >4, having smoked at least 15 cigarettes a day 12 months prior to enrollment, and had no intention to quit during the study. Participants were not included if they had any earlier experience with transcutaneous electric nerve stimulation (TENS), TEAS, EA or manual acupuncture or if they used any nicotine replacement therapies or “stop smoking” therapies during the 3-month period prior to enrollment in the present study. Subjects with pacemakers, a history of chronic medical conditions, any acute illness or reported alcohol/drug abuse were also not included. Participants were required to sign a written informed consent to participate and to stop smoking from the Friday evening 18:00 h until Saturday evening 20:00 h, for exactly 26 h. Participants were paid SGD100 for their participation in each experiment. Both study protocols were approved by the NUH Ethics Committee and implemented in the NUH Clinical Trial Unit (CTU).

### 2.3. Transcutaneous Electrical Acupoints Stimulation

The TEAS device (HANS LH-202, Huawei Co. Beijing, China) is a dual-channel acupoint nerve stimulator with two pairs of constant current electric output. The frequency of the output stimulation is an alternating dense-and-disperse mode, where the 2 Hz (0.6-ms pulse width) stimulation is alternated with 100 Hz stimulation (0.2-ms pulse width) automatically, each lasting for 3 s. The device is connected to the subject via four carbon-pad adhesive electrodes at four acupoints. They are Hegu (LI4, located at the mid-point of the second metacarpus on the radial side), and Laogong (PC8, mid-way between the second and third metacarpus on the palmal side, where one's middle finger falls when one makes a fist) in the same hand to form an electric circuit. The other two points are: Nei Guan (PC6, 2*″* above the palm, between the palmaris longus and flexor carpi radialis tendon), and Wai Guan (TE5, 2*″* above the skin crease on the back of the wrist, between the ulnar and radius) located on the opposite forearm to complete a circuit. Earlier observations revealed no adverse effects of these stimulations, except for very rare instances of skin irritation, such as redness beneath or around the electrodes, which usually disappeared in 1-2 h.

In the first study (Experiment 1), a specially designed control-TEAS device was used for the control group, which capped the output intensity to 5 mA, even if the investigator selected the protocol-required intensity of 10 mA.

In the second study (Experiment 2), the same control-TEAS device (capped at 5 mA) was used but was combined with a specific-switching device programmed to switch the stimulation on for 3 min and off for 7 min (the i5 mA group), or totally blocking the stimulation from reaching the subject (the 0 mA group). A flashing signal indicated that the treatment was in progress; this was added to enhance the blinding further.

In both the studies, the hands of the subject were placed in an opaque box when receiving stimulation, so that the participants and the investigators could not observe the amplitude of the finger movements and guess the relative intensity he/she was receiving.

### 2.4. Guess Test

Moreover, to ascertain the validity of the double-blind, all the participants were asked to complete a guess test at the end of each session which comprised of the following questions:

(1) “Do you believe that your TEAS unit was functioning properly”? The answers ranged from “I am certain it was working properly” to “I am certain it was not working”, and scored from 0 to 4.

(2) “If your answer is that you had absolutely no idea whether the unit was functioning or not, what would you guess”? The answer could be either “Functioning properly” or “Not functioning properly”.

At the end of each session, the investigators also completed an observer guess test containing the same aforementioned two questions.

### 2.5. Outcome Measures

The primary outcome measure was the total score of smoking urges as assessed by the 10-item Questionnaire of Smoking Urges-Brief (QSU-Brief) [27]. The QSU-Brief is derived from the multidimensional Questionnaire of Smoking Urges (QSU) [28]. The QSU-Brief has been demonstrated to be sensitive to urges occurring during acute cigarettes abstinence [27]. This questionnaire has been validated [27] in both a laboratory setting, and an outpatient smoking cessation clinic. The QSU-Brief can be completed in 2 min. Participants respond by ticking the desired score between “strongly agree” and “strongly disagree” on a seven-point Likert scale. The total score ranges from 10 to 70, and the subscores for each factor range from 5 to 35. The QSU questionnaire was administered at time 0 before participants stopped smoking and then at 2, 14, 18, 22, 24 and 26 h after the last cigarette was smoked.

Systolic blood pressure (SBP), diastolic blood pressure (DBP) and pulse rate (PR) were recorded by using GE Marquette Dash 3000 Bedside Patient Monitoring System. Body temperature (°C), (measured orally using the Terumo Digital Clinical Thermometer, Model C0402), and respiration rate (breaths per minute, BPM) were recorded each time just before the participants completed the QSU questionnaire.

Carbon monoxide (CO) levels in the expired air using a CO monitor (Minismokerlyzer, Bedfont Scientific Limited, UK) were measured before the participants stopped smoking, and during abstinence at 14, 18, 22, 24 and 26 h after the last cigarette was smoked. Blood samples for plasma cotinine determinations were drawn at baseline and at 14 and 22 h after smoking was stopped. Plasma cotinine concentrations were determined by chemiluminescence using Immulite 2000 (Diagnostic Products Corporation, USA). The reference limit is <25 ng ml^−1^ for non-smokers. The assay sensitivity is 5 ng ml^−1^ with a within-run precision of 4.0–9.9%.

### 2.6. Study Procedures

The screening/enrollment was performed for not >10 days before the participants started the study. The study was initiated on a Friday evening at 18:00 h, and lasted until Saturday evening 20:00 h.

For both the studies, 18–20 participants were assessed during each session. All participants were instructed to smoke their last cigarettes upon their arrival at the study center, and then not to smoke again until the end of the study. Participants were allowed to go back home for the night.

### 2.7. Statistical Analyses

The categorical variables of demographics and clinical characteristics were summarized by frequency, whereas the continuous variables were summarized by arithmetic mean and standard deviation (SD). The frequency of categorical parameters between the groups was compared with Pearson's chi-squared test, and Fisher's exact test was used if the expected count by cell was less than five. Continuous variables between the two independent groups were compared with the Mann-Whitney non-parametric test.

All the responses from the 10-question QSU-Brief were summed up as a single score (minimum score, 10; maximum score, 70) for each participant at each predefined time points, and was compared between treatment arms as a function of time. On separate factor analysis of QSU scores, according to Cox et al. [27], QSU scores from questions 1, 3, 6, 7 and 10 were summed up as Factor 1 (positive reinforcement), and the other questions (2, 4, 5, 8 and 9) of the QSU-Brief were summed up as Factor 2 (negative reinforcement).

The variations in the mean QSU scores across time points by each treatment arm were plotted. Exploratory graphical presentation suggested that a growth curve quadratic model might be used to fit the observed QSU scores as a function of time. Each interaction term was tested and the covariate, *Time* and quadratic term, *Time* ^2^, were significant univariate terms, which were included in the fitted model. All the models were adjusted by the covariate: age. Gender was not a significant covariate term in the quadratic model, and was not included in the fitted models. Growth-curve quadratic model was used to analyze the trend of both Factors 1 and 2 between arms as a function time. Likewise, all models were adjusted by covariate age. The predefined time points were entered as *t* = 0, 1, 2,… 6 for time 0, 2, 14, 18, 22, 24 and 26 h after the last cigarette was smoked. Missing values were not imputed.

All analyses were performed on SAS (Version 9.1, SAS Institute Inc., Cary, NC, USA). A two-sided *P* < .05 was considered significant.

## 3. Results

### 3.1. Demographic and Smoking Characteristics of the Participants

The relevant data in Experiments 1 and 2 are depicted in Table 1. Around half of the participants were of Chinese origin while the others were of Indian, Malay or Indonesian origin. There were no statistically significant differences between the groups in either study regarding gender, race, age, number of cigarettes smoked per day, duration of smoking and FTND score (Table 1). In addition, there was no significant difference in the expired CO and plasma cotinine levels between the study groups, either at baseline or during smoking abstinence.

### 3.2. Inclusions and Exclusions

Three participants in the 5 mA arm, one participant in the 10 mA arm of Experiment 1 decided to stop the study. Two participants from each arm of Experiment 2 did not return to the center overnight after the second QSU, and one participant of the 0 mA group did not continue the treatment after the fourth QSU assessment. A total of nine minor adverse events were reported by the participants. In the 10 mA arm, one patient reported a runny nose and coughing 14 h after the last cigarette, and one patient experienced giddiness after blood drawn at time 0, and numbness in a finger after 2 h, and this subject decided not to complete the study. In the i5 mA group, three participants reported a runny nose and one reported insomnia. In the 0 mA group, one participant reported a runny nose after 14 h and did not complete the study, one reported giddiness, and one reported numbness in the left hand.

### 3.3. Analysis of the Guess Tests

Analysis performed in Experiments 1 and 2 showed that the participants were unable to distinguish which stimulations they received (Fisher's exact test: *P* = .44 and *P* = .66, resp.). Observer guess scores were similar across groups in both Experiments 1 and 2 (Fisher's exact test: *P* = 1.0 and *P* = .8, resp.). Thus, the efficacy analyses could be performed.

### 3.4. Experiment 1

**Figure 1:**
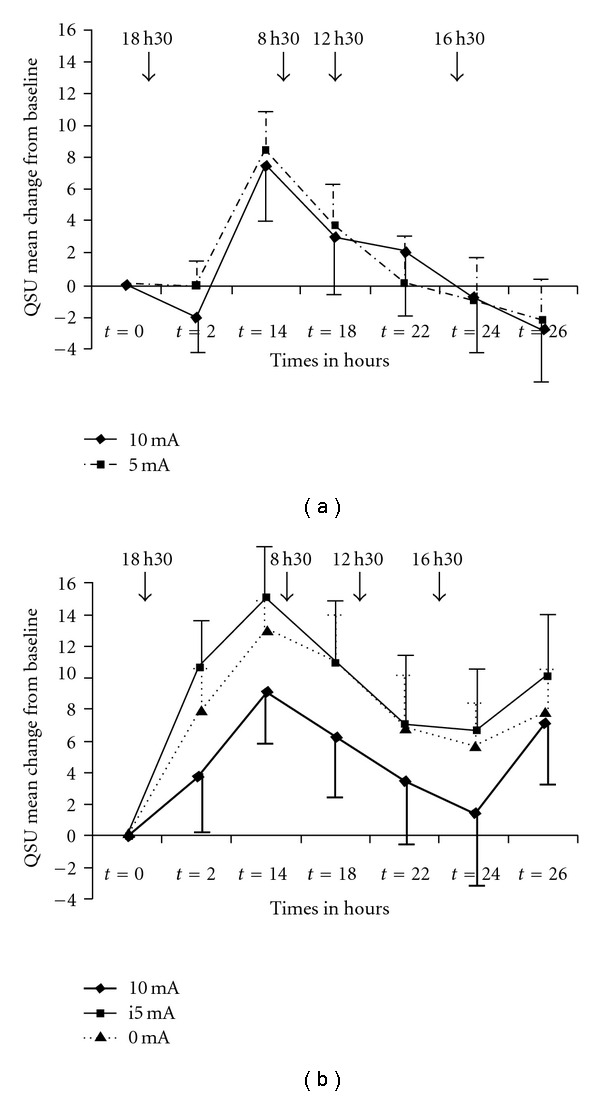
Mean QSU changes from baseline in Experiments 1 (a) and 2 (b). Arrows indicate TEAS. Error bars indicate SEM.

The average baseline QSU scores were 42.6 ± 15.20 and 36.8 ± 14.96 (mean ± SD) in the 10 and 5 mA group, respectively, showing no significant difference. The fitted quadratic model showed that there was no significant condition by time interaction (*P* = .89 and .96, for first- and second-order interaction terms, resp.). Further, no difference occurred either for Factor 1 (*P* = .53) or for Factor 2 (*P* = .29) (data not shown) of the QSU-Brief. As seen in Figure 1, (a) shows mean QSU change scores from baseline.

### 3.5. Experiment 2

The average QSU scores for the baseline of 10, i5 and 0 mA groups were 43.62 ± 17.37, 42.5 ± 14.64 and 39.29 ± 16.44, respectively, showing no statistically significant difference. There was no main effect of condition, and the fitted quadratic model showed that there was no significant condition by time interaction (*P* = .19 and .24, for first- and second-order interaction terms, resp.). As seen in Figure 1, (b) shows mean QSU change scores from baseline. Moreover, analysis of Factors 1 and 2 showed no main effect.

### 3.6. Exploratory Analyses in Subjects with FTND Score ≥4

In the exploratory analyses of Experiment 2, interventions were dichotomized into control arm consisting of 0 and i5 mA (*n* = 37) and active arm consisting of 10 mA (*n* = 21). Statistical analysis of the first- and second-order interactions showed that there was a tendency toward significant condition by time interaction (*P* = .09 and .11, resp.), the increase in QSU during abstinence being somewhat less in the 10 mA condition than in the control condition.

There was no significant condition by time interaction for Factor 1 (*P* = .12 and .2 for first- and second-order interaction terms, resp.) but a significant condition-by-time interaction occurred for Factor 2 (*P* = .005 and .017 for first- and second-order interaction terms, resp.).

### 3.7. Exploratory Analyses in Subjects with FTND ≥5

**Figure 2:**
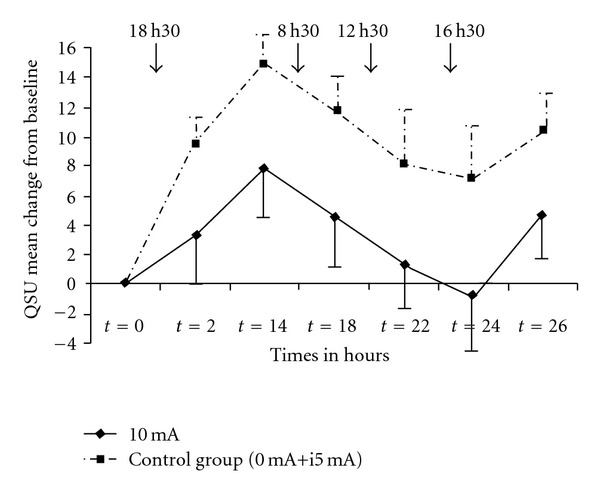
Mean QSU changes from baseline in Experience 2. The two placebo conditions (0 mA and i5 mA) were merged into control condition and compared to the 10 mA condition. Subjects with FTND* ≥5 were included. Arrows indicate TEAS. Error bars indicate SEM.

When only subjects with FTND scores ≥5 were selected (*n* = 35 for control arm and *n* = 20 for the 10 mA arm), a significant first-order condition by time interaction was observed (*P* = .02) (Figure 2). The second-order interaction showed a tendency toward significant activity (*P* = .056). A significant condition-by-time interaction for Factor 2 was also found (*P* = .017 and .054, for first- and second-order interaction terms, resp.), but not for Factor 1. In both the experiments, no difference was observed between any of the groups regarding systolic, diastolic blood pressure, pulse rate or temperature.

## 4. Discussion

### 4.1. Experimental Design

#### 4.1.1. The Design of the Control Group

The difficulty of designing a control group for acupuncture lies in the fact that acupuncture by itself produces sensations and subjective feelings, the so-called “Deqi” sensation [29]. To provide the subject with a credible acupuncture sensation, yet limiting the therapeutic effect, a minimal stimulation modality is often used [30]. The key point resides in the choice of the optimal intensity for the control stimulation. The TEAS device used in the present study delivers constant current electrical stimulation. The threshold intensity for producing a tingling sensation was found to be 5 mA in most cases. Therefore, in the first study, we used the threshold intensity of 5 mA for control, and doubled the threshold value to 10 mA as the active intensity. However, the results showed that the changes in the degree of the urge to smoke were similar. This could be interpreted to mean that (i) none of the two treatments was effective, or (ii) a minimal stimulation was already effective.

#### 4.1.2. The Design of a Device Used as Placebo/Control Treatments

Eliminating and reducing any bias is a major goal of controlled studies [31–33]. After an exhaustive review of available publications on blinding physical therapies, we determined that while there is no room for further reducing the intensity of the threshold stimulation, and at the same time keeping the tingling sensation, we tried to reduce the length of stimulation. By inserting a brake device between the output sockets of the TEAS and the skin electrodes, we were able to control the timing of the stimulation in a 3 min on and 7 min off schedule (designated as Intermittent 5 or i5 mA group). The subjects in this group could clearly feel the tingling sensation from time to time, which may have helped convince the subject that real electrical stimulation is delivered to the stimulation sites. The inconvenience of a placebo electrical stimulation (0 mA) is that the subject can guess with a relatively high likelihood that there is no stimulation (no skin sensations); the inconvenience for a proxy of a placebo electrical stimulation (i5 mA) is that even an intermittent and a very weak stimulation may have some biological effect. Being unsure of the validity of these two alternatives, we decided to design two placebo groups: 0 and i5 mA. The guess tests showed that the subjects could not distinguish between the interventions. Given the strong placebo effect, it is evident from Figure 1(a) that the effect induced by i5 mA was almost identical with that produced by the blank control (0 mA), suggesting that this mode of stimulation was too weak to produce a measurable biological effect. Moreover, the main statistical analysis showed that the estimated coefficients of the 0 and i5 mA arms were very close together and far from the estimated coefficients of the 10 mA arm. The results obtained from the 0 and i5 mA group were thus merged to form a single control group.

#### 4.1.3. Features to Promote Blinding of Subjects and Investigators

These features include the use of “placebo” TEAS devices that are visually identical to active devices, exclusion of patients with earlier TEAS/acupuncture/TENS experience, use of identical protocol/visit schedule, avoidance of treatment discussion between participants and clinicians, and the use of visual signals. In addition, the subjects' hands were hidden in a box so that neither the investigators nor the subjects could assess the amplitude of the subjects' finger movements due to electrical stimulations, and finally each participant was explained that “each of the three treatment modalities was set with different levels of intensity, frequency, duration or administration mode, and that some of these parameters such as intensity are more stimulatory than others”. Hence, they would probably feel some sensation with high-intensity stimulations and no sensation with high-frequency stimulations which could nevertheless be as effective. This design proved successful, as the guess test showed that the double-blinding was not compromised.

### 4.2. Mode of Acupuncture

Considering the future potential application of the technique by the smokers at home, we chose the TEAS instead of manual needling.

Concerning the criteria of choosing the four acupoints, the Hegu point is known to be the most powerful point to produce an analgesic effect via the increase of release of opioid peptides, which are released in the CNS [19], whereas Neiguan, a strategic point of the pericardial meridian is known to produce a calming or anxiolytic effect according to traditional Chinese medicine. When EA or TEAS is used instead of manual needling, it is advisable to use the close-by point to complete an electric circuit rather than to use the point in the opposite extremity, thereby letting the current pass through the cardiac region. Repeated use of acupuncture is needed for most of the therapeutic interventions. In the treatment of heroin addiction with TEAS, it was used three times a day in the first 5 days of drug abstinence, followed by twice a day for the following 5 days, and ending with once a day for the rest of the detoxification period of 15 days [22]. From a clinical point of view, repeated use seems to be necessary for effective symptom control. However, too frequent a use, for example, 4–6 times a day, will unavoidably lead to the development of tolerance [34]. Therefore, a three-times-a-day schedule was used in the present study.

### 4.3. Exploratory Analysis

Participants for these two studies were included only if they had FTND scores >4, because the urge to smoke (or craving) increases with higher tobacco dependence [35]. Our results showed that in this population, TEAS did not antagonize the increase in urge to smoke. However, when analyses were limited to participants with FTND ≥5, 10 mA, TEAS did antagonize abstinence-induced increase in the urge to smoke. A similar pattern has been observed for the antinociceptive effect of TEAS in rats. Standardized acupuncture procedure applied to a normal rat produces an increase of the nociceptive threshold by 80–100%, an effect lasting only for a period of <1 h [36]. However, in a rat subjected to neurogenic hyperalgesia (by the ligation of the lumbar five and six dorsal spinal nerve roots), one session of electro-acupuncture for 30 min produced a suppression of hyperalgesia for as long as 24 h [37]. Thus, the efficacy of TEAS seems to depend on the degree of disturbance of homeostasis: the higher the deviation from the normal level, the more effective is the acupuncture intervention.

### 4.4. The Effect of TEAS on Factors 1 and 2

The effect of TEAS to antagonize the urge to smoke concerned only Factor 2 of the QSU, suggesting that TEAS is more effective in reducing an anticipation of relief from negative effect with an urgent desire to smoke during smoking abstinence. Based on our systemic study of the neurochemical mechanisms of acupuncture therapy in animal models [19], it was evident that TEAS produces a frequency-dependent release of opioid peptides in the CNS [19, 38]. Low frequency (2 Hz) TEAS increases the release of enkephalins, endorphins and endomorphin in the brain to interact with the *μ*- and *δ*-opioid receptors, 100 Hz increases the release of dynorphin in the spinal cord to interact with the kappa opioid receptors, whereas 2/100 Hz mode has been shown to accelerate the release of all four kinds of endogenous opioid peptides [19]. Since chronic nicotine has been shown to inhibit proopiomelanocortin (POMC) gene expression, and thus probably, biosynthesis of *β*-endorphin [39], the activation of endorphin release by TEAS [38] may play a role in ameliorating the deficiency of endorphin. On the other hand, activation of central kappa receptors is a powerful suppressor of the withdrawal syndrome in rats made dependent on morphine [22, 40], and in opiate addicts [41], although it remains unclear whether it also works in relieving nicotine withdrawal. In a recent study [42], it was reported that in rats dependent on nicotine, withdrawal of nicotine resulted in an anxiety-like behavior. This psycho-neural disorder can be effectively reduced by acupuncture. The authors attributed this effect to the negative modulatory effect of acupuncture to the expression of corticotrophin-releasing factor (CRF) in the amygdala. Indeed, the CRF mRNA levels in the amygdala were significantly higher in the nicotine withdrawal group compared to the control group, and acupuncture brought the CRF mRNA level down to the control level.

As to the lack of significant effect of TEAS on Factor 1 (strong desire and intention to smoke, with smoking perceived as rewarding) of the QSU, one has to consider that the subjects were not allowed to smoke during the 26 h of the study, and lack of smoking cannot induce positive reinforcement, so it is not surprising that the TEAS intervention did not affect the positive reinforcement.

### 4.5. Limitations

The subjects enrolled in this study were mainly of Asian origin. Thus, the present results may not be generalized to smokers of other ethnic origins.

The exploratory analysis showed that only the subgroup of more dependent smokers showed significant antagonism in the urge to smoke by TEAS, but this analysis was not planned, which weakens its strength.

Any earlier alcohol or any illicit drug consumption was not assessed (and controlled for) by structured interview, which would potentially lead to lower homogeneity of the population studied.

Finally, we assessed only a surrogate of smoking cessation (urge to smoke). The efficacy of TEAS on smoking cessation should be assessed in therapeutic trials.

## 5. Conclusions

The results obtained in the present study indicate that application of TEAS on the skin is capable of antagonizing the urge to smoke in dependent smokers. Based on these preliminary findings, it seems warranted to assess the efficacy of TEAS in larger populations of dependent smokers, and to plan further for clinical trials for smoking cessation.

## Figures and Tables

**Table 1:** Demographic and smoking characteristics of subjects participating in Experiment 1 and 2.

	Experiment 1		Experiment 2		
Treatment condition	5 mA	10 mA	0 mA	i5 mA	10 mA
	(*N* = 20)	(*N* = 20)	(*N* = 17) Placebo	(*N* = 20) A proxy of placebo	(*N* = 21)
Gender (*N*, %)					
Female	0 (0)	2 (10)	2 (12)	7 (35)	6 (29)
Male	20 (100)	18 (90)	15 (88)	13 (65)	15 (71)
Age (years), Mean ± SD	30.8 ± 6.47	30.5 ± 8.78	25.9 ± 4.75	23.5 ± 3.58	24.7 ± 4.85
Number of cigarettes per day Median (range)	20 (15–35)	18.5 (15–28)	19 (15–28)	18.3 (15–25)	17.0 (15–23)
Years of smoking Median (range)	14 (5–31)	13.5 (6–38)	9.0 (1–23)	7.5 (4–13)	8.0 (5–28)
FTND Median (range)	5.5 (4–8)	5.5 (4–8)	7.0 (4–8)	7.0 (4–9)	6.0 (4–8)
